# Effectiveness of workers’ general health examination in Korea by health examination period and compliance: retrospective cohort study using nationwide data

**DOI:** 10.1186/s40557-017-0160-5

**Published:** 2017-02-17

**Authors:** Huisu Eom, Jun-Pyo Myong, Eun-A Kim, Bohwa Choi, Soon Woo Park, Young Joong Kang

**Affiliations:** 1 Occupational Safety and Health Research Institute, Korea Occupational Safety and Health Agency, 400, Jongga-ro, Ulsan, 44419 Jung-gu Republic of Korea; 2 Department of Occupational and Environmental Medicine, Seoul St. Mary’s Hospital, College of Medicine, Catholic University of Korea, Seoul, Republic of Korea

**Keywords:** Workers’ General Health Examination, Health examination period, Compliance, Cardio-cerebrovascular disease

## Abstract

**Background:**

Our study evaluated the effectiveness of the Workers’ General Health Examination by health examination period and compliance.

**Methods:**

A retrospective cohort of the health examination participants in 2006 (baseline year: *N* = 6,527,045) was used. We identified newly occurring cardio-cerebrovascular disease over 7 years (from 2007 to 2013). After stratification by age, sex, and national health insurance type, we identified 7 years’ cumulative incidence of cardio-cerebrovascular disease by health examination compliance and estimated its relative risk by health examination period and compliance.

**Results:**

The compliant group presented a lower cumulative incidence of cardio-cerebrovascular disease than the non-compliant group; this result was consistent across sex, working age (40s and 50s), and workplace policyholder. Relative risk of cardio-cerebrovascular disease by health examination period (1 and 2 years) showed statistically significant results in ischemic heart disease for male participants. Of men in their 40s, office workers (over a 2-year period) presented statistically higher relative risk of ischemic heart disease than non-office workers (over a 1-year period: 1.03; 95% confidence interval, 1.02–1.03). However, there were no consistent results in ischemic cerebrovascular disease and hemorrhagic cerebrovascular disease for men or cardio-cerebrovascular disease for women.

**Conclusion:**

A 1-year period of Workers’ General Health Examinations in non-office workers had a more significant prevention effect on ischemic heart disease than a 2-year period in office workers among working age (40s–50s) men. It is, however, necessary to consider that prevention of cardio-cerebrovascular disease can be partially explained by their occupational characteristics rather than by health examination period.

## Background

Periodic health examinations are systematic scheduled screenings performed for more than one risk factor or disease in more than one organ system [1]. The purpose of periodic health examinations in Korea is to detect target diseases at an early stage by screening the asymptomatic general population and ultimately reducing the mortality rate of the target diseases [2]. One of the periodic health examinations currently being conducted in Korea, the Workers’ General Health Examination (WGHE) has a similar purpose. The purpose of WGHEs is to improve labor productivity and worker’s health protection by finding ordinary or occupational diseases at an early stage and offering participants appropriate follow-up action [3]. The health status of workers is a main factor affecting company productivity. Therefore, it is important to effectively perform WGHEs, not only for corporate profit and worker’s health status but also for national health [4, 5].

The effectiveness of periodic health examinations was estimated in several studies. In Japan, comprehensive periodic health examinations have occurred for many years. Cardiovascular disease specific mortality [6] was lower among participants than non-participants in Japanese health check-ups; overall mortality [6–8] was also lower among the participating group. Prevention of cardiovascular disease is also the main target of WGHEs in Korea [2].

Conversely, in Korea, study of periodic health examination effectiveness, especially WGHEs, has been insufficient. Although several studies analyzed periodic health examination effectiveness in Korea, most of them only targeted National General Health Examinations (NGHEs), one of the periodic health examination now performed by the National Health Insurance System (NHIS). One simulation analysis by NHIS of NGHE cost-effectiveness [9] showed 0.76 increase in quality adjusted life year per diabetes patient who participated in NGHE. Other reports showed statistically lower mortality [10, 11] and cardio-cerebrovascular disease risk [10] among NGHE participants than non-participants. However, no study considered health examination period (1 vs. 2 years) as an independent variable in the analysis.

Distinguishing WGHEs and NGHEs was not meaningful in the previous studies, because WGHEs were replaced by NGHE results after 1995 [12]. However, WGHEs retains its own characteristics under the labor act from the perspective of workers’ health [12] and occupational health surveillance [13]. Furthermore, WGHE periods are classified into 1-year (non-office worker) and 2-year (office worker) for workplace policyholders; the influence of period on WGHEs has not yet been studied. Therefore, effectiveness analysis of WGHEs and study of this unverified variable is necessary.

The present study evaluated WGHE effectiveness by health examination period and compliance. For this purpose, we created a retrospective cohort based on the health examination participants in 2006 and identified newly occurring cardio-cerebrovascular disease (CCVD) during 7 years (from 2007 to 2013).

## Methods

### Study population

The responsibility for workplace policyholders’ health examinations was transferred from the Ministry of Labor to Ministry of Welfare in 1995 by *the Occupational Safety and Health Act* [12, 14]. Therefore, the targets of this study, WGHE examinees (now performed by the Ministry of Labor), are the same as workplace policyholders with NGHEs (now performed by the Ministry of Welfare). Thus, we regarded workplace policyholders’ NGHE results (from 2002 to 2006) as WGHEs. Moreover, we were also provided regional policyholders’ NGHE data during the same years and combined them in the analysis.

**Fig. 1:**
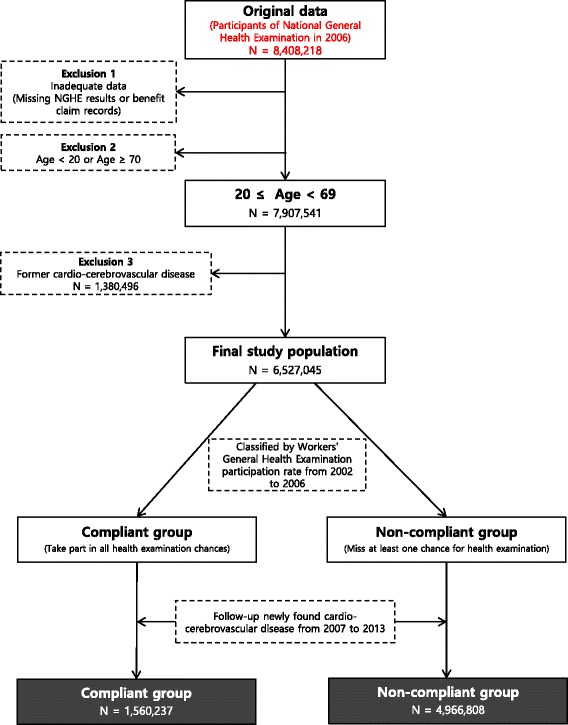
Flow chart of study population

We designed a retrospective cohort study based on 2006 NGHE results. From the total NGHE participants in 2006 (*N* = 15,053,761), 8,408,218 participants were identified (Fig. 1). We excluded participants with inadequate NGHE results or NHIS benefit claim records. Participants aged over 70 or less than 20 were also excluded. Participants with past CCVD in the NHIS benefit claim record were excluded to identify newly occurring CCVD. The final study population (*N* = 6,527,045) were confirmed. Then, we stratified this study population by age, sex, and national insurance type. In this procedure, we classified national insurance types into workplace and regional policyholders. Workplace policyholders were divided into office and non-office workers for more detail. Public officers and public educational personnel and staff were excluded from the analysis because of few examinees.

### Definition of health examination compliance

The study population in the present research was divided into two subgroups, compliant and non-compliant, by WGHE participation rate. The classification criterion was whether examinees participated in all health examination chances during 5 years (from 2002 to 2006). When examinees participated in all health examination chances, they were classified into the compliant group. Conversely, when examinees omitted at least one health examination chance, they were classified as non-compliant (Table 1). Because health examination periods differed by national health insurance type, classification criterion for the compliant group varied by national health insurance type. Non-office workers were defined as compliant when they participated in 5 total health examination chances (from 2002 to 2006) because their health examination period was 1 year. Office workers and regional policyholders were defined as compliant when they participated in 3 total health examination chances because their health examination period was 2 years.

**Table 1:** Definition of compliant group by participation rate in Workers’ General Health Examination

	Compliant group		Non-compliant group
	Non-office worker^a^	Office worker and self-employed^b^	Any national insurance type^c^
Year			
2002	Participation	Participation	Miss at least one chance for health examination
2003	Participation		
2004	Participation	Participation	
2005	Participation		
2006 (Base year)	Participation	Participation	

^a^Participate in five total health examination chances from 2002 to 2006

^b^Participate in three total health examination chances in 2002, 2004, and 2006

^c^Missing at least one chance for health examination

### Outcome ascertainment

WGHE effectiveness was assessed by the number of newly occurring CCVD (ischemic heart disease, ischemic cerebrovascular disease, and hemorrhagic cerebrovascular disease) after 2006. Each CCVD case was identified by matching participants’ datasets with NHIS’ benefit claim records through participant’s temporary identification numbers that omit any personal information. To identify newly occurring CCVD, we excluded participants who had CCVD before 2006. After that, we identified newly occurring CCVD from 2007 to 2013. However, diseases caused by other reasons than vessel ischemia or hemorrhage (such as recurrent disease or diseases by trauma) were not considered in this analysis. The International Classification Disease 10 (ICD 10) code of CCVD is presented in Table 2.

**Table 2:** ICD-10 code of target disease

ICD-10 code^a^	
Ischemic heart disease	
I20	Angina pectoris
I21	Acute myocardial infarction
I25	Chronic ischemic heart disease
Ischemic cerebrovascular disease	
I63	Cerebral infarction
I64	Stroke, not specified as hemorrhage or infarction
I69.3	Sequelae of cerebral infarction
I69.4	Sequelae of stroke, not specified as hemorrhage or infarction
G45	Transient cerebral ischemic attacks and related syndromes
Hemorrhagic cerebrovascular disease	
I60	Subarachnoid hemorrhage
I61	Intracerebral hemorrhage
I62	Other nontraumatic intracranial hemorrhage
I69.0	Sequelae of subarachnoid hemorrhage
I69.1	Sequelae of intracerebral hemorrhage
I69.2	Sequelae of other nontraumatic intracranial hemorrhage

^a^International Classification of Diseases-10

### Statistical analysis

This study was performed in the following order. As a first step, we divided the study population into two subgroups by health examination compliance (Table 3) and identified each group’s 7-year cumulative CCVD incidence (Table 4). This presented possible differences in the preventive effects of health examination between the compliant and non-compliant groups. We calculated cumulative incidence in percentages by the fraction of number of newly developed CCVD over number of WGHE participants in baseline year 2006.

**Table 3:** Baseline characteristics of study subjects by health examination compliance (N = 6,527,045)

		Compliant group^a^ (*N* = 1,560,237)		Non-compliant group^b^ (*N* = 4,966,808)	
Characteristic	Total	N	(%)	N	(%)
Sex					
Male	3,563,547	1,031,161	(28.9%)	2,532,386	(71.1%)
Female	2,963,498	529,076	(17.9%)	2,434,422	(82.1%)
Age category (years)					
20–29	129,792	78,305	(60.3%)	51,487	(39.7%)
30–39	933,018	405,873	(43.5%)	527,145	(56.5%)
40–49	2,041,281	447,599	(21.9%)	1,593,682	(78.1%)
50–59	1,552,300	335,015	(21.6%)	1,217,285	(78.4%)
60–69	1,870,470	293,444	(15.7%)	1,577,026	(84.3%)
National insurance type					
Office worker	428,032	198,213	(46.3%)	229,819	(53.7%)
Non-office worker	1,392,354	903,968	(64.9%)	488,386	(35.1%)
Self-employed	4,706,475	458,055	(9.7%)	4,248,420	(90.3%)

^a^Participate in all health examination chances from 2002 to 2006

^b^Missing at least one chance for health examination from 2002 to 2006

**Table 4:** Cumulative incidence rate (7 years) of cardio-cerebrovascular disease by health examination compliance

Disease	Male						Female					
	Compliant group^b^			Non-compliant group^c^			Compliant group^b^			Non-compliant group^c^		
Age and National insurance type	Subjects	Cases^a^	(%)	Subjects	Cases^a^	(%)	Subjects	Cases^a^	(%)	Subjects	Cases^a^	(%)
Ischemic heart disease												
40s												
Non-office worker	265,236	26,167	(9.87%)	148,658	16,055	(10.8%)	55,396	4,170	(7.53%)	26,707	2,047	(7.66%)
Office worker	51,875	5,933	(11.44%)	65,510	8,604	(13.13%)	8,132	581	(7.14%)	10,357	787	(7.6%)
Self-employed	19,171	2,524	(13.17%)	676,348	69,611	(10.29%)	47,789	5,536	(11.58%)	666,102	63,002	(9.46%)
50s												
Non-office worker	138,224	25,701	(18.59%)	75,595	14,982	(19.82%)	39,794	6,019	(15.13%)	18,655	2,921	(15.66%)
Office worker	18,490	3,952	(21.37%)	34,071	8,742	(25.66%)	1,778	310	(17.44%)	4,141	781	(18.86%)
Self-employed	41,434	9,144	(22.07%)	480,593	91,265	(18.99%)	95,295	19,511	(20.47%)	604,230	110,136	(18.23%)
Ischemic cerebrovascular disease												
40s												
Non-office worker	265,236	10,295	(3.88%)	148,658	6,363	(4.28%)	55,396	2,338	(4.22%)	26,707	1,042	(3.9%)
Office worker	51,875	2,014	(3.88%)	65,510	2,899	(4.43%)	8,132	283	(3.48%)	10,357	416	(4.02%)
Self-employed	19,171	1,122	(5.85%)	676,348	30,745	(4.55%)	47,789	3,013	(6.3%)	666,102	33,053	(4.96%)
50s												
Non-office worker	138,224	12,870	(9.31%)	75,595	7,714	(10.2%)	39,794	3,348	(8.41%)	18,655	1,699	(9.11%)
Office worker	18,490	1,656	(8.96%)	34,071	3,594	(10.55%)	1,778	141	(7.93%)	4,141	376	(9.08%)
Self-employed	41,434	5,240	(12.65%)	480,593	51,301	(10.67%)	95,295	11,810	(12.39%)	604,230	64,021	(10.6%)
Hemorrhagic cerebrovascular disease												
40s												
Non-office worker	265,236	1,631	(0.61%)	148,658	1,265	(0.85%)	55,396	308	(0.56%)	26,707	143	(0.54%)
Office worker	51,875	294	(0.57%)	65,510	523	(0.8%)	8,132	45	(0.55%)	10,357	61	(0.59%)
Self-employed	19,171	188	(0.98%)	676,348	7,850	(1.16%)	47,789	383	(0.8%)	666,102	5,930	(0.89%)
50s												
Non-office worker	138,224	1,708	(1.24%)	75,595	1,127	(1.49%)	39,794	337	(0.85%)	18,655	220	(1.18%)
Office worker	18,490	188	(1.02%)	34,071	465	(1.36%)	1,778	17	(0.96%)	4,141	45	(1.09%)
Self-employed	41,434	717	(1.73%)	480,593	9,449	(1.97%)	95,295	1,253	(1.31%)	604,230	8,949	(1.48%)

^a^Cumulative incidence of cardio-cerebrovascular disease during 7 years, 2007 to 2013

^b^Participate in all health examination chances from 2002 to 2006

^c^Missing at least one chance for health examination from 2002 to 2006

For the second step, we identified relative CCVD risk by health examination compliance (Tables 5 and 6). For this purpose, we targeted non-office workers who have the most chances for health examinations (1-year period) and calculated the relative risk of the compliant group (reference: non-compliant group). The results were classified by sex and age (40s and 50s).

**Table 5:** Relative risks for cardio-cerebrovascular disease by health examination compliance and period among male participants

Male			Ischemic heart disease			Ischemic cerebrovascular disease			Hemorrhagic cerebrovascular disease		
Age and national insurance type	period	Subjects	Cases	RR^e^	(95% CI)	Cases	RR^e^	(95% CI)	Cases	RR^e^	95% CI
40s											
Non-office worker											
Non-compliant group^a^		148,658	16,055	1.00	(ref)	6,363	1.00	(ref)	1,265	1.00	(ref)
Compliant group^**b**^		265,236	26,167	0.94	(0.93 to 0.95)	10,295	0.94	(0.92 to 0.96)	1,631	0.82	(0.79 to 0.86)
National insurance type											
Non-office worker^c^	1 year	265,236	26,167	1.00	(ref)	10,295	1.00	(ref)	1,631	1.00	(ref)
Office worker^d^	2 year	51,875	5,933	1.03	(1.02 to 1.03)	2,014	1.00	(0.99 to 1.01)	294	0.99	(0.97 to 1.01)
Self-employed^d^	2 year	19,171	2,524	1.03	(1.02 to 1.03)	1,122	1.04	(1.03 to 1.04)	188	1.04	(1.02 to 1.06)
50s											
Non-office worker											
Non-compliant group^a^		75,595	14,982	1.00	(ref)	7,714	1.00	(ref)	1,127	1.00	(ref)
Compliant group^b^		138,224	25,701	0.95	(0.94 to 0.96)	12,870	0.94	(0.92 to 0.96)	1,708	0.89	(0.85 to 0.93)
National insurance type											
Non-office worker^c^	1 year	138,224	25,701	1.00	(ref)	12,870	1.00	(ref)	1,708	1.00	(ref)
Office worker^d^	2 year	18,490	3,952	1.02	(1.02 to 1.03)	1,656	1.00	(0.99 to 1.00)	188	0.98	(0.96 to 0.99)
Self-employed^d^	2 year	41,434	9,144	1.05	(1.05 to 1.06)	5,240	1.09	(1.08 to 1.10)	717	1.09	(1.07 to 1.12)

^a^Missing at least one chance for health examination from 2002 to 2006

^b^Participate in total health examination chances from 2002 to 2006

^c^Only compliant group of non-office workers was selected: representing 1 year period health examination

^d^Only compliant group of office workers and self-employed was selected: representing 2 year period health examination

^e^Relative risk and 95% confidence interval for cardio-cerebrovascular disease were estimated by SAS syntax: PROC GENMOD

**Table 6:** Relative risks for cardio-cerebrovascular disease by health examination compliance and period among female participants

Female			Ischemic heart disease			Ischemic cerebrovascular disease			Hemorrhagic cerebrovascular disease		
Age and national insurance type	period	Subjects	Cases	RR^e^	(95% CI)	Cases	RR^e^	(95% CI)	Cases	RR^e^	95% CI
40s											
Non-office worker											
Non-compliant group^a^		26,707	2,047	1.00	(ref)	1,042	1.00	(ref)	143	1.00	(ref)
Compliant group^b^		55,396	4,170	0.99	(0.95 to 1.02)	2,338	1.06	(1.00 to 1.11)	308	1.03	(0.90 to 1.18)
National insurance type											
Non-office worker^c^	1 year	55,396	4,170	1.00	(ref)	2,338	1.00	(ref)	308	1.00	(ref)
Office worker^d^	2 year	8,132	581	1.00	(0.98 to 1.00)	283	0.98	(0.96 to 0.99)	45	1.00	(0.96 to 1.04)
Self-employed^d^	2 year	47,789	5,536	1.28	(1.25 to 1.31)	3,013	1.24	(1.20 to 1.28)	383	1.21	(1.11 to 1.31)
50s											
Non-office worker											
Non-compliant group^a^		18,655	2,921	1.00	(ref)	1,699	1.00	(ref)	220	1.00	(ref)
Compliant group^b^		39,794	6,019	0.97	(0.94 to 1.00)	3,348	0.94	(0.91 to 0.98)	337	0.81	(0.73 to 0.89)
National insurance type											
Non-office worker^c^	1 year	39,794	6,019	1.00	(ref)	3,348	1.00	(ref)	337	1.00	(ref)
Office worker^d^	2 year	1,778	310	1.01	(1.00 to 1.01)	141	1.00	(0.99 to 1.00)	17	1.01	(0.98 to 1.03)
Self-employed^d^	2 year	95,295	19,511	1.31	(1.28 to 1.34)	11,810	1.38	(1.33 to 1.42)	1,253	1.39	(1.27 to 1.53)

^a^Missing at least one chance for health examination from 2002 to 2006

^b^Participate in total health examination chances from 2002 to 2006

^c^Only compliant group of non-office workers was selected: representing 1 year period health examination

^d^Only compliant group of office workers and self-employed was selected: representing 2 year period health examination

^e^Relative risk and 95% confidence interval for cardio-cerebrovascular disease were estimated by SAS syntax: PROC GENMOD

Finally, we identified relative CCVD risk by health examination period (1-year vs. 2-year; Tables 5 and 6). For this purpose, we calculated the relative risk of the 2-year group (regional policyholders and office workers) based on the 1-year group (non-office workers). Participants in this analysis included only the compliant group. In this procedure, all relative risks were calculated after stratification of study population by sex and age (40s and 50s). Relative risk (RR) and 95% confidence interval (CI) for CCVD were estimated by SAS syntax: PROC GENMOD and SAS ver. 9.3 was used in all statistical analysis.

## Results

### Baseline characteristics of study population

A total of 6,527,045 participants were enrolled in the cohort study population from the original 2006 NGHE data (Table 3). The non-compliant group was larger than the compliant group. The proportion of men in the compliant group was larger than the proportion of women. The age group with the highest proportion in the compliant group was the 20s; the others were lower in the order: 30s, 40s, and 50s. The proportion of those aged in the 60s in the compliant group was very low. Among the three national insurance types, non-office workers were the highest proportion of the compliant group followed by office workers and regional policyholders. Of regional policyholders, participants in all health examination chances for 5 years were relatively few (9.7%) compared to other national insurance types.

### Distribution of cardio-cerebrovascular disease by compliance

The distribution about cumulative CCVD incidence by health examination compliance is presented in Table 4. The cumulative incidence of ischemic heart disease (IHD) in the compliant group was lower than non-compliant group among non-office male workers in their 40s. Office worker results were similar. In addition, male non-office workers who had 1-year chances for health examinations showed a lower incidence IHD gap between the compliant and non-compliant groups (0.93%) than office workers with 2-year chances for health examinations (1.69%). Conversely, regional policyholders in the compliant group showed higher IHD incidence than in the non-compliant group. The above results were consistent in men and women in both the 40s and 50s age groups.

Ischemic cerebrovascular disease (ICVD) results were similar to IHD. The cumulative incidence in the compliant group was lower than non-compliant group in non-office male workers in their 40s; this result was consistent in office workers. In addition, the ICVD incidence gap between compliant and non-compliant groups male non-office workers (1-year period; 0.4%) was lower than office workers (2-year period; 0.55%). Conversely, regional policyholders’ cumulative ICVD incidence in the compliant group (5.85%) was higher than the non-compliant group (4.55%).

Hemorrhagic cerebrovascular disease (HCVD) incidence in the compliant group of office and non-office workers was low. The low HCVD incidence rate among the compliant group of workplace policyholders was the same as the IHD and ICVD incidence rate analyses. In regional policyholders, by contrast, HCVD incidence in the compliant group was lower than the non-compliant group in a different pattern than IHD or ICVD incidence.

### Relative risk of cardio-cerebrovascular disease by compliance

Relative CCVD risk by health examination compliance is presented in Tables 5 and 6. Male non-office workers in the compliant group showed lower CCVD risk than in the non-compliant group (Table 5). This result for male non-office workers was consistent with other diseases, ICVD and HCVD, and also consistent across both age groups (40s and 50s). However, relative CCVD risks for women in the compliant group did not show statistically significant results (Table 6).

### Relative cardio-cerebrovascular disease risks between 1-year and 2-year groups

The relative risks of CCVD by health examination period (1- and 2-year) are presented in Tables 5 and 6. In this analysis, only IHD for male participants showed statistically significant results, whereas women did not present consistent results. Male office workers in their 40s (2-year period) presented statistically higher relative IHD risk than non-office workers (1-year period). The result was the same for men in their 50s. However, there were no consistent results in ICVD and HCVD for men and CCVD for women. Relative risks of IHD, ICVD, and HCVD in regional policyholders (2-year period), by contrast, were statistically higher than non-office workers (1-year period). These results were consistent in both sexes (Tables 5 and 6).

## Discussion

“Health examination compliance” is a new variable reflecting the participation rate of periodic health examinations for several years. Using the consistency of annual participation as a health measure has never been attempted in past research. Previous research related to periodic health examinations in Korea usually analyzed single-year participation [9, 10, 15]. It is possible to assess not only single-year health effects of periodic health examinations but also its multiyear health effects when we use health examination compliance as an analysis variable. We identified that the compliant group has lower cumulative CCVD incidence than the non-compliant group (Table 4) consistently in both sexes and workplace policyholders. Moreover, we identified that the relative CCVD risk in the compliant group was statistically lower than the non-compliant group for male non-office workers (Table 5). Therefore, we suggest that health examination compliance positively affects CCVD prevention among workplace policyholders.

Further analysis compared the cumulative CCVD incidence between health examination periods (1-year vs. 2-year). We identified that the relative IHD risk of male office workers (2-year period) was statistically higher than that of male non-office workers (1-year period; Table 5). Although the analysis was limited in that the results did not show statistical significance or consistent results in both sexes and diseases, participants who received 1-year health examination showed better preventive effects than 2-year health examination for IHD in working age (40s–50s) men. There are several studies of periodic health examination effectiveness; however, a consensus remains lacking. One meta-analysis using only randomized controlled trials published from 1963 to 1999 (14 trials) revealed that periodic health examination has no beneficial effect on total mortality (RR = 0.99, 95% CI: 0.95 to 1.03) and cardiovascular mortality (RR = 1.03, 95% CI: 0.91 to 1.17) [1]. Another systematic review using 23 observational studies and 10 randomized controlled trials published from 1973 to 2004 also reported that periodic health examinations may be related with increased use of preventive medical service and reduced patient worry, but additional research data is needed to estimate its long term benefit [16]. Conversely, several investigations conducted in Japan showed that periodic health examinations had positive effects on total mortality (hazard ratio [HR] = 0.74, 95% CI: 0.62 to 0.88 [6]; HR = 0.70, 95% CI: 0.56 to 0.88 [7]; HR = 0.83, 95% CI: 0.69 to 0.99 [8]) and cardiovascular disease mortality (HR = 0.65, 95% CI: 0.44 to 0.95) [6] for men. The effectiveness of mass periodic health examinations is still controversial because of the difficulty of large clinical trials with periodic health examination [6]. There are no studies using health examination period as an independent variable to our knowledge despite the controversial results.

The relative CCVD risks between office and non-office workers showed subtle differences ranging from 0.93 to 1.03 (Tables 5 and 6). These results may be caused by a large study population (*N* = 6,527,045) not the effect of health examinations. However, such a subtle difference might represent a meaningful result from the perspective of public health and prevention. Moreover, male non-office workers’ (1-year period) CCVD incidence gaps between the compliant and non-compliant groups were lower than office workers’ (2-year period; Table 4 for both IHD and ICVD). Thus, giving more participation chances for health examinations can narrow health effect gaps between subgroups classified by compliance.

Two perspectives are possible for why differences in effectiveness of health examinations were not identified in either ICVD or HCVD but were for IHD. One possibility is that ICVD and HCVD actually do not differ in effects by health examination period, unlike IHD. Another possibility is disease characteristics such as peak age and etiology of IHD and stroke (including both ICVD and HCVD). Although both IHD and ICVD have the same cause (arteriosclerosis), each disease shows differences in peak age and incidence as vessel ischemia from different organs (heart and brain) [17]. The peak age for IHD is the 50s–60s and 36% of IHD patients are under 45 years old [17, 18]. Conversely, ICVD occurs at a relatively older age than IHD. ICVD is rare before age 40. ICVD prevalence doubles every 10 years after age 55, so the highest prevalence (about 27%) is identified at over 80 years [19]. Therefore, a 7-year follow up period might be insufficient time for ICVD to detect effectiveness of health examinations, because ICVD occurs at relatively older ages than IHD (50s–60s). Further, HCVD’s pathophysiology itself is fundamentally different from IHD. Blood vessel rupture is the main cause of HCVD. In addition, the incidence of HCVD is 24.6 per 100,000 person-years; this value is one-tenth of IHD’s incidence (434 per 100,000 person-years) [17, 19]. HCVD’s relatively low incidence makes it difficult to draw statistically significant results, while IHD incidence analysis presented significant results.

Statistical significance was not consistent for women’s CCVD incidence by health examination period. Two perspectives are also possible for this result. One possibility is that health examination has no preventive effect for women; another possibility is the difference in disease epidemiology between the sexes. IHD occurs 10 to 20 years later in women than men and IHD occurrence in women is rare before menopause [20]. Women’s occurrence age for stroke is also later than men and the incidence rate is 33% lower than men [17, 21]. Etiology of ischemic stroke also differs between the sexes. Large vessel atherosclerotic stroke and associated coronary and peripheral artery diseases are more common in men and cardiac embolism-related stroke is more common in women [22]. Therefore, a 7-year follow up period might be insufficient time to detect IHD and stroke in women because women’s CCVD incidence is lower than men and occurrence is later than men. Further research with long-term follow up periods can determine differences of health examination effectiveness between the sexes.

Relative CCVD risks in regional policyholders with 2-year health examination periods were higher than non-office workers with 1-year periods; these results were statistically significant in both sexes. However, careful attention is needed in this analysis. Selection bias by the healthy worker effect (HWE) [23] is possible between non-office workers and regional policyholders. Healthy workers have greater potential to initiate their career in better companies and continue to work for longer than unhealthy workers [24]. Therefore, unhealthy workplace policyholders are likely to be retired from their workplace and to be regional policyholders. As a result, it is possible that health status differences occurred between the two selected groups; workplace policyholders may be relatively healthier than regional policyholders. Health examination compliance may be confounded by HWE in the same manner as well. Healthy workers are more likely to get health examinations stably than unhealthy workers due to better working environment [24]. Therefore, the variable, health examination compliance, may be confounded by healthy workers’ stable participation in health examination.

The 1-year period health examination had more preventive effects on ischemic heart disease than the 2-year period. It is necessary to identify the reasons for the difference in CCVD risk by period in further studies. Although there were several reports that described the mechanism of periodic health examination effectiveness, most were about its possible benefits. Participant’s poor health habits (e.g., smoking, alcohol drinking, irregular meals, no regular exercise) might be changed through medical counseling during periodic health examinations and periodic result notifications [1]. Identifying abnormal results (e.g., high blood pressure, glucose, cholesterol) in the early stages of disease also may lead to early intervention and health management [1]. It is also possible that WGHEs had a positive effect on medical accessibility by improving the delivery of medical intervention; the more health examination opportunities they have, the more chance for medical intervention they have [16].

This study has some specific limitations. The first is HWE, mentioned in detail above. The second is inaccuracy of benefit claim records and health examination results in NHIS. In this research, we utilized ICD 10 codes from NHIS benefit claim records instead of hospital medical records. Benefit claim records request treatment charges to NHIS. Over-rated diagnosis coding is possible to avoid cutbacks in benefit claim records [10] and diagnosis can be inaccurate in some instances [10, 25]. Third, the 7-year follow up period was insufficient to successfully evaluate WGHEs. This limitation can be one reason that statistically significant results were not consistently shown in ICVD, HCVD, and women, as previously mentioned. Finally, there are possible confounding factors between office and non-office workers. Although we stratified the two subgroups by sex, age, and national insurance type, several confounders in evaluation of health examinations remain. Socioeconomic status such as income, education, and residential district, and lifestyles such as smoking, alcohol consumption, and exercise are well known determinants of health examinations [26–29]. In addition, there are several reports showing that white collar workers have greater tendency to chronic diseases than blue collar workers. Several studies reported that the risk of chronic diseases such as dyslipidemia, hypertension and metabolic syndrome were higher in white collar than blue collar workers [30, 31]; this result was consistent even though they worked in the same work place [30]. The reasons chronic disease risks differ by job style were analyzed in several studies; sedentary work of white collar workers can influence worker’s health status negatively by physical inactivity [30, 32]. WGHE results include lifestyle questionnaire data (e.g., smoking, alcohol drinking, regular meals, regular exercise, etc.), but we did not analyze the questionnaire data in this study. Other significant variables such as working hours and level of sedentary work were not available from the questionnaire data.

Because of above limitations including possible confounding factors, our study is limited in comparing office and non-office workers. However, definitions classifying office and non-office workers in our database are somewhat different from those of blue and white collar workers in the above articles. According to *The Occupational Health and Safety Act*, sedentary workers who work in the same territory or are exposed to similar occupational environments to manual workers (e.g., sedentary workers whose offices adjoin their firm’s factory) are classified as non-office workers, although their job style is only paperwork. As a result, our data (e.g. office and non-office worker) did not reflect past job classification categories (e.g., white and blue collar or non-manual and manual worker). This is one possible reason our study lacked some above confounding factors between white and blue collar workers. It is necessary to consider the job classifications in this study to interpret the present results.

Despite these limitations, our research has several valuable findings. First, this study was performed with a real dataset provided by NHIS, not by simulation techniques. More than 6 million participants were the target of analysis using nationwide health examination data. Initial studies usually used simulation techniques; research using real datasets were not attempted [9, 15, 33]. Since then, Yoon et al. [10] analyzed a real dataset and Jee et al. [11] adjusted a cohort study design to NHIS nationwide data. Second, we presented a 5-year continuous participation rate, not just a single-year participation rate, by defining the new concept of “health examination compliance.” Several reports presented single-year participation rates [14]; however, our research presented multiyear participation rates for the first time. In 2006, 77% of workplace policyholders participated in NGHEs [12] but the compliant group who participated in all health examination chances for 5 years (2002 to 2006) was only 24% of the total cohort group (Table 3). Single- and continuous-year participation rates need to be included in further analysis of periodic health examinations.

However, the most important point is our evaluation of WGHE effectiveness by health examination period for the first time. WGHE periods in Korea differ between office (2-year) and non-office (1-year) workers. There are non-office workers who participate in health examinations every year and office workers who participate in it biennially; thus, it is possible that their health effects differ. Previous studies only analyzed health examination effectiveness by participation and did not consider health examination periods as a descriptive variable [9–11]. For the first time, we showed that 1-year WGHE periods in non-office workers had more significant prevention effect for IHD than 2-year periods in office workers among working age (40–50s) men. However, prevention of cardio-cerebrovascular disease can be partially explained by their occupational characteristics rather than their health examination period. Our study result should influence national health policy and support the necessity of further research. Additional studies adjusting variables such as lifestyle and socioeconomic status of participants and long-term follow-ups are needed based on this study.

## Conclusion

Our study showed that 1-year periods for Workers’ General Health Examinations in non-office worker had more preventive effect on ischemic heart disease than 2-year periods in office workers among working-age (40s–50s) men. In addition, the compliant group showed a lower 7-year cumulative cardio-cerebrovascular disease incidence than the non-compliant group. However, prevention of cardio-cerebrovascular disease can be partially explained by their occupational characteristics rather than their health examination period. Efforts to conduct more systematic effect evaluation on Worker’s General Health Examinations should be made by using adjusting various determinants of health examination and long-term follow-ups, based on this study result.

## Abbreviations

CCVD: Cardio-cerebrovascular disease
CI: Confidence interval
HCVD: Hemorrhagic cerebrovascular disease
HR: Hazard ratio
HWE: Healthy worker effect
ICD 10: International classification disease 10
ICVD: Ischemic cerebrovascular disease
IHD: Ischemic heart disease
NGHE: National general health examination
NHIS: National health insurance service
RR: Relative risk
WGHE: Workers’ general health examination
